# Characterization of the Fungal Community in Fritillariae Cirrhosae Bulbus through DNA Metabarcoding

**DOI:** 10.3390/jof8080876

**Published:** 2022-08-19

**Authors:** Jingsheng Yu, Wenjuan Zhang, Yujie Dao, Meihua Yang, Xiaohui Pang

**Affiliations:** 1 Institute of Medicinal Plant Development, Chinese Academy of Medical Sciences & Peking Union Medical College, Beijing 100193, China; 2 Institute for Control of Chinese Traditional Medicine and Ethnic Medicine, National Institutes for Food and Drug Control, Beijing 102629, China

**Keywords:** collection areas, DNA metabarcoding, fungi, traditional Chinese medicine

## Abstract

Fritillariae Cirrhosae Bulbus (FCB) is a well-known and precious traditional Chinese medicine with a medicinal history spanning thousands of years. In recent years, it has been reported that fungal and mycotoxin contamination influenced the safety and quality of FCB. It is essential to systematically study the fungal community for the early warning of fungal and mycotoxin contamination in this herb. A total of 15 FCB samples were collected from five provinces in China, and the fungal communities in the FCB samples were analyzed via amplifying the internal transcribed spacer 2 region through the Illumina Miseq PE300 platform. Furthermore, we compared the differences in fungal community in five groups based on collection areas. Results showed that Ascomycota (41.58–99.66%) and Mucoromycota (0–57.42%) were dominant at the phylum level. Eurotiomycetes (8.49–63.93%), Eurotiales (8.49–63.53%), and Aspergillaceae (8.49–63.51%) were the most abundant at the class, order, and family levels. *Aspergillus* (8.49–63.41%), *Rhizopus* (0–57.42%), *Fusarium* (0–22.81%), *Cladosporium* (0.16–9.14%), and *Alternaria* (0.06–17.95%) were the main genera in FCB samples. A total of 34 fungal taxa were identified at the species level, including five potentially toxigenic fungi namely *Penicillium brevicompactum*, *P. citrinum*, *P. oxalicum*, *Trichothecium roseum*, and *Aspergillus restrictus*. The differences in fungal community between the five groups were observed. Our findings provide references for the safe utilization and quality improvement of FCB.

## 1. Introduction

Fritillariae Cirrhosae Bulbus (FCB), which is known as a precious traditional Chinese medicine (TCM), has been applied in the clinic for thousands of years. FCB exhibits efficient expectorant, antitussive, and antiasthmatic activities according to the Chinese Pharmacopoeia [1,2]. The growth of FCB requires strict environmental conditions. Most FCB plants are distributed in high-altitude areas with altitudes between 3000 and 5000 m, such as Qinghai province, Tibet, and western Sichuan province [3]. Thus, the annual production of FCB is low. The market demand for FCB has been remaining at a high level in recent years due to its medical application in the treatment of COVID-19, which increased the prices of this TCM [4]. Given its abundant nutrients, FCB becomes a proper host for the growth of fungi. Fungal contamination in FCB has been studied previously. Zheng et al. reported the fungal contamination in 45 TCM samples, and isolated three strains from FCB, including two *Penicillium* strains and one *Eurotium* strain [5]. The mycotoxin contamination, which was synthesized by some potential mycotoxin-producing fungi under proper conditions, has also been reported in FCB. Han et al. investigated 35 mycotoxins in 60 TCM samples, and positively detected three mycotoxins in FCB samples namely Chaetoglobosin A (2.1 μg/kg), cyclopiazonic acid (36.1 μg/kg), and gliotoxin (2.3 μg/kg) [6]. Therefore, studying fungal contamination to provide references for the early prevention of mycotoxin contamination in FCB is essential.

In recent years, increasing numbers of studies have reported the fungal contamination in TCMs. Yu et al. reported that *Aspergillus*, *Penicillium*, and *Fusarium* were the common fungal genera that contaminated various TCMs [7]. Chen et al. analyzed fungal and mycotoxin contamination in fresh and dry Liquorice samples collected from China, and results showed that *Aspergillus* and *Penicillium* were dominant among all genera. Meanwhile, they detected ochratoxin A contamination in 6 out of 12 Liquorice samples with levels ranging from 12.99 to 39.03 μg/kg [8]. A report by Jiao et al. assessed the effect of *Fusarium solani* and *Fusarium oxysporum* on the growth and ginsenoside metabolites of American ginseng, demonstrating that two *Fusarium* strains inhibited the growth of American ginseng, and even reduced ginsenoside levels [9]. Kong et al. isolated 124 fungal strains from 24 functional food and spice samples and identified three potential mycotoxin-producing fungi including *Aspergillus* *niger*, *A. flavus*, and *A. ochraceus* [10]. Previous studies demonstrated that fungal contamination became a concern that affected the quality and safety of TCM. Meanwhile, different TCMs are contaminated by different kinds of fungi. Therefore, an efficient method for the investigation of fungal contamination in TCMs is needed.

The concept of DNA metabarcoding was explained by Porter and Hajibabaei [11]. Specifically, DNA metabarcoding is a sensitive method that identifies multiple species simultaneously in a single sample by amplifying a representative DNA region. Compared with traditional identification methods, DNA metabarcoding identifies more fungal taxa with low relative abundance [12]. In addition, many uncultured microorganisms can be identified through DNA metabarcoding, which provides a comprehensive view of the microbial community [13]. Thus, DNA metabarcoding has been applied in various fields in recent years. Fang et al. evaluated the effect of Berberine on postmenopausal anxiety through the modulation of the gut microbiome. Their results showed that the gut microbiome played an important role in the treatment of postmenopausal anxiety [14]. Matsuoka et al. monitored the seasonal changes in fungal community in forest streams and found that fungal community exhibited regular annual changes that could provide information for the variation in microbiome in terrestrial ecosystems [15]. In addition, DNA metabarcoding has been used to analyze the fungal community in several seed and fructus TCMs [16,17,18,19].

To the best of our knowledge, the fungal community in FCB has not been previously reported. In the present study, we collected FCB samples from the main producing areas, and analyzed the diversity and composition of fungal community using DNA metabarcoding. Furthermore, the differences in fungal community in FCB samples collected from various areas were compared. The aim of this study is to analyze the fungal community of FCB and compare the differences in the fungal composition and diversity between groups based on collection areas, providing a scientific basis for the targeted prevention of fungal contamination.

## 2. Materials and Methods

### 2.1. Sample Collection

A total of 15 FCB samples were collected from the local herbal markets in Tibet (Xizang in Chinese, *n* = 3), Anhui (*n* = 3), Hebei (*n* = 3), Sichuan (*n* = 3), and Qinghai Provinces (*n* = 3), which were the main production areas or trade markets. All samples were derived from the dried bulb of FCB. Each sample was collected with approximately 250 g and stored in a sterile paper bag. All FCB samples were transported to the Institute of Medicinal Plant Development. The samples were stored at −20 °C until the DNA extraction. Table 1 lists the detailed information of each FCB sample.

### 2.2. DNA Extraction and PCR Amplification

Approximately 3.3 g of FCB samples were weighed, and then transferred into 50 mL sterile centrifuge tubes containing 15 mL of 1 × PBS buffer (Beijing Solarbio Science and Technology Co., Ltd., Beijing, China). The mixture was shaken for six minutes and filtered through four layers of sterile gauze. All filtrates were centrifugated at 12,000 rpm for 26 min to collect fungal strains for total DNA extraction. The fungal DNA was extracted from FCB samples by using an EZNA^®^ soil DNA kit (Omega Bio-tek, Norcross, GA, USA) according to the manufacturer protocols. We amplified the internal transcribed spacer 2 (ITS2) sequences by using the universal primers ITS3/ITS4 [20]. The amplification conditions were as follows. Initial denaturation at 95 °C for 3 min; 35 cycles of denaturation at 95 °C for 30 s, annealing at 55 °C for 30 s, elongation at 72 °C for 45 s, followed by a final extension at 72 °C for 10 min. The DNA products were checked for quality through 2% agarose gel electrophoresis. The purified DNA amplicons were pooled in equimolar quantities, and sequenced through Illumina Miseq PE300 platform (Illumina, San Diego, CA, USA).

### 2.3. Bioinformatic Analysis

Raw data containing chimeric and low-quality reads were filtered to obtain high-quality sequences using Fastp software, (v.0.19.6, https://github.com/OpenGene/fastp, accessed on 10 November 2021) and Flash software (v.1.2.11, https://ccb.jhu.edu/software/FLASH/index.shtml, accessed on 10 November 2021). The clean sequences were clustered into operational taxonomic units (OTUs) with 97% similarity by Uparse (v. 7.0.1090, http://www.drive5.com/uparse/, accessed on 16 November 2021). Each OTU was classified at various levels that ranged from the genus to the phylum level by Qiime (v.1.9.1, http://qiime.org/install/index.html, accessed on 16 November 2021). In order to ensure the identification accuracy, each OTU was verified through the manual BLAST search of the International Nucleotide Sequence Database Collaboration. The raw sequencing data were uploaded to the National Center for Biotechnology Information Sequence Read Archive database with the accession numbers SAMN24255245-SAMN24255259. Mothur version v.1.30.2 (http://www.mothur.org/, accessed on 16 November 2021) was used to calculate the alpha diversity index, including Chao, Shannon, and Good’s coverage. The indexes reflected the diversity and abundance of fungi in the FCB samples. The significant differences in the indexes were analyzed by using Student’s T test. The rarefaction curve, column diagram, and Venn graph were constructed using R software v.3.3.1 (available online https://www.r-project.org/, accessed on 16 November 2021). The beta diversity analysis was performed through hierarchical clustering, principal co-ordinates analysis (PCoA), and non-metric multidimensional scaling analysis (NMDS) using Qiime and R software v.3.3.1 (available online https://www.r-project.org/, accessed on 16 November 2021). Significant differences among groups were analyzed through Kruskal–Wallis H test. The LEfSe analysis was performed for biological statistical difference using LEfSe software (http://huttenhower.sph.harvard.edu/galaxy/root?tool_id=lefse_upload, accessed on 18 November 2021). The network figure was constructed by Networkx.

## 3. Results

### 3.1. Analysis of the Fungal Diversity in FCB Samples

The ITS2 sequences in 15 FCB samples were amplified successfully, and the quality of the PCR products met the requirement for Illumina sequencing. The results showed that 965,875 clean sequences (260–460 bp) were obtained after sequencing. The rarefaction curve demonstrated that the sequencing depth was sufficient to reflect the fungal community in each sample (Figure S1). We normalized the data to a depth of 17,276 sequences (the minimum sample sequence number) per sample, and it was sufficient to estimate the fungal community diversity. The highest and lowest numbers of sequences were observed in FCBQH2 (104,378 sequences) and FCBXZ3 (50,350 sequences). All sequences were clustered into 529 OTUs (Table S1). The Venn diagram illustrated that 214 OTUs were shared between five groups based on collection areas. Meanwhile, each group had its own unique OTU, and the unique OTU numbers were as follows: FCBAH (38 OTUs), FCBQH (38 OTUs), FCBHB (111 OTUs), FCBSC (93 OTUs), and FCBXZ (35 OTUs, Figure S2). Three alpha diversity indices were estimated to assess the alpha fungal community diversity in the 15 FCB samples. The Chao index results revealed that the highest and lowest fungal community abundances were observed in FCBHB3 and FCBAH2. The fungal community diversity was reflected by the Shannon index. Our results showed that FCBAH1 and FCBAH3 showed the highest and lowest fungal diversity. The Good’s coverage analysis showed that the indices of all FCB samples were >99.9%, indicating that these indices were sufficient to reflect the depth of the fungal community in samples (Table 2).

### 3.2. Analysis of the Fungal Community in the FCB Samples

**Figure 1:**
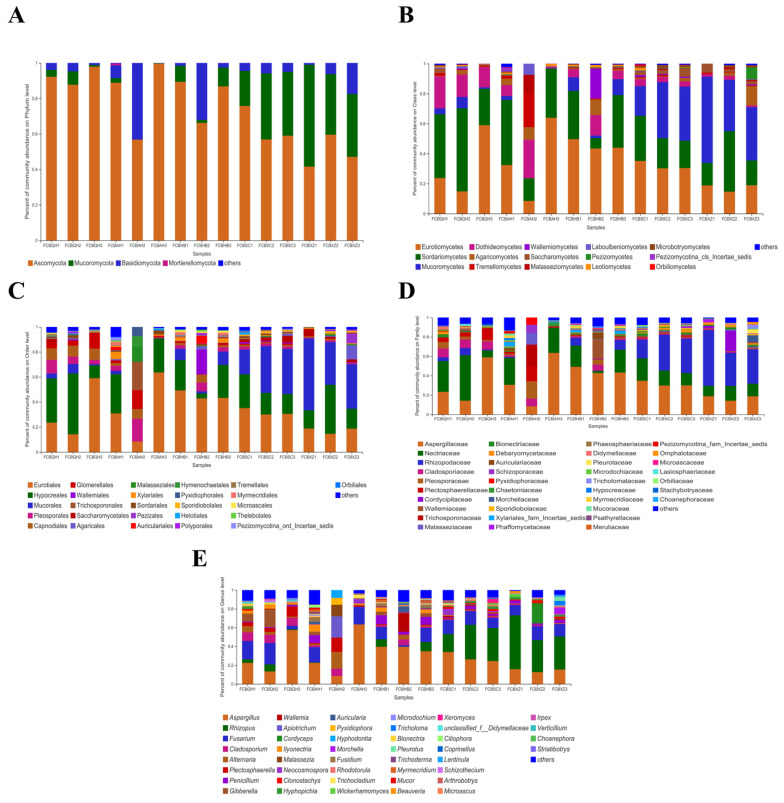
Fungal community composition in 15 FCB samples at the (**A**) phylum level; (**B**) class level; (**C**) order level; (**D**) family level; and (**E**) genus level. Genera, whose relative abundances were over 1% in at least one sample, were displayed, and others represent the sum total of other genera whose relative abundances were less that 1%.

The OTUs were classified at various taxonomic levels. The fungal community in the FCB samples was further studied at the phylum, class, order, family, and genus levels. At the phylum level (Figure 1A), Ascomycota (41.58–99.66%) was dominant, followed by Mucoromycota (0–57.42%), Basidiomycota (0.15–43.20%), and Mortierellomycota (0–10.66%). At the class level (Figure 1B), Eurotiomycetes (8.49–63.93%), Sordariomycetes (6.97–55.44%), and Mucoromycetes (0–57.42%) exhibited higher numbers in FCB samples than other classes. The order composition analysis showed that Eurotiales (8.49–63.53%), Hypocreales (0–48.56%), and Mucorales (0–57.42%) had the highest relative abundances among all orders (Figure 1C). Further taxonomic analysis revealed that Aspergillaceae (8.49–63.51%), Nectriaceae (0–46.89%), and Rhizopodaceae (0–57.42%) predominated at the family level (Figure 1D). *Aspergillus* (8.49–63.41%), *Rhizopus* (0–57.42%), *Fusarium* (0–22.81%), *Cladosporium* (0.16–9.14%), and *Alternaria* (0.06–17.95%) were the dominant genera (Figure 1E). At the species level, a total of 34 fungal taxa were identified (Table S2), among which five were potentially toxigenic namely *Penicillium brevicompactum*, *P. citrinum*, *P. oxalicum*, *Trichothecium roseum*, and *Aspergillus restrictus*.

### 3.3. Comparison of the Differences in Fungal Community in Five FCB Groups Based on Collection Areas

**Figure 2:**
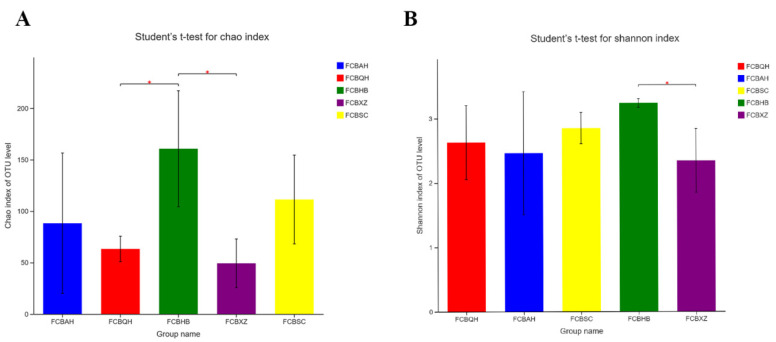
(**A**) Statistically significant different Chao indexes of the five FCB groups; (**B**) statistically significant different Shannon indices of the five FCB groups. The abscissa is the group name and the ordinate is the exponntial average index of each group. * represents *p* ≤ 0.05.

**Figure 3:**
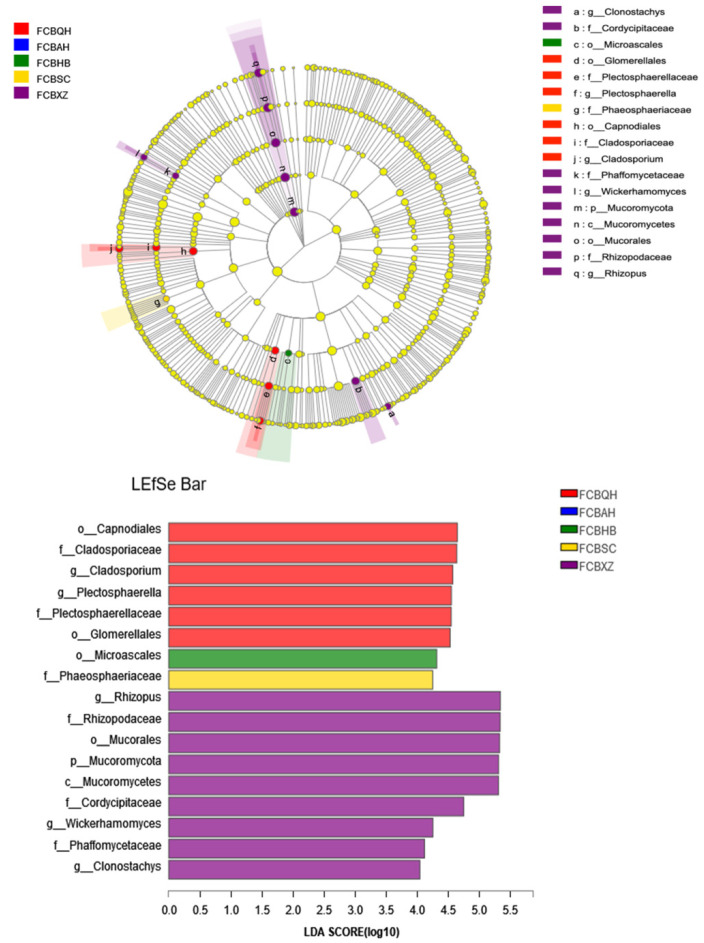
LEFSe analysis of differences in fungal community ranging from the genus level to the phylum level. Different color nodes denote the microbial groups significantly enriched in the corresponding groups and that had significant influence on the differences between groups.

**Figure 4:**
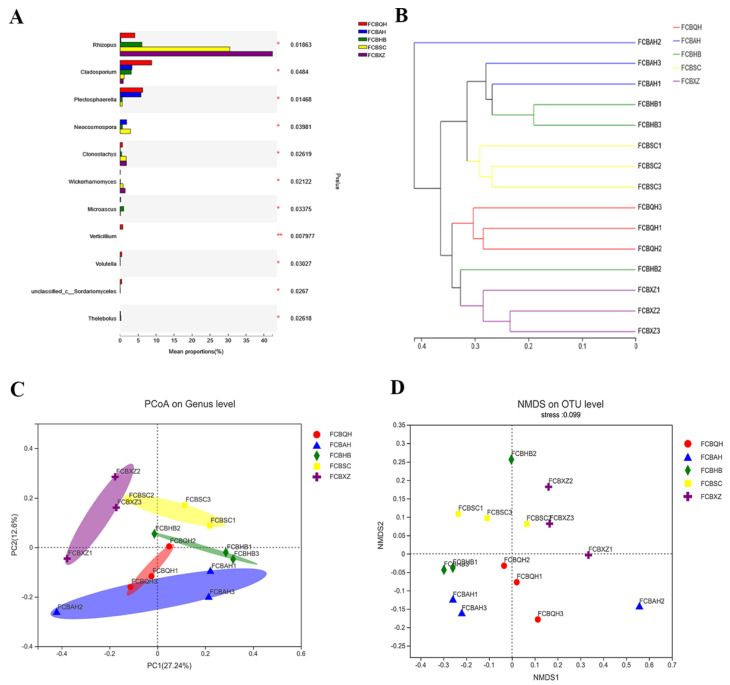
(**A**) Significant difference test on FCB groups via the Kruskal−Wallis H test; (**B**) hierarchical clustering analysis of the fungal community in five FCB groups at the OTU level; (**C**) PCoA plot at the genus level generated through the Bray−Curtis; (**D**) NMDS plot at the OTU level estimated with unweighted unifrac.

The 15 FCB samples were divided into five groups based on the collection areas. Significant difference analysis showed that the FCBHB (158.67) and FCBXZ groups (49.33) had the highest and lowest Chao indexes. The statistically differences were observed between the FCBHB and FCBQH groups (*p* = 0.043), as well as between the FCBHB and FCBXZ groups (*p* = 0.034, Figure 2A). Shannon analysis illustrated that the FCBHB (3.24) and FCBXZ groups (2.34) showed the highest and lowest index, respectively, and represented the highest and lowest fungal community diversity among five groups. The statistic difference was observed between the FCBHB and FCBXZ groups (*p* = 0.036, Figure 2B). The differences in fungal community were compared through LEfSe analysis (Figure 3). Our results illustrated differences in the fungal community in the five groups at various taxonomic levels. The relative abundances of Capnodiales (order level), Glomerellales (order level), Cladosporiaceae (family level), Plectosphaerellaceae (family level), *Cladosporium* (genus level), and *Plectosphaerella* (genus level) were higher in the FCBQH group than in other groups, whereas the relative abundance of Microascales (order level) in the FCBHB group was the highest among all groups. The FCBXZ group had higher numbers of Mucoromycota (phylum level), Mucoromycetes (class level), Mucorales (order level), Rhizopodaceae (family level), Cordycipitaceae (family level), Phaffomycetaceae (family level), *Rhizopus* (genus level), *Wickerhamomyces* (genus level), and *Clonostachys* (genus level) than the other groups, and the FCBSC group had the highest number of Phaeosphaeriaceae (family level) among the five groups. As depicted in Figure 4A, the significant difference test between five groups revealed that the relative abundance of *Rhizopus* was significantly higher in FCBXZ than those in the other groups. *Cladosporium*, *Verticillium*, and *Volutella* showed higher relative abundances in the FCBQH group than in the other groups. The relative abundance of *Plectosphaerella* in the FCBQH and FCBAH groups was higher than that in the other groups. The hierarchical clustering tree generated at the OTU level showed that the samples in the five groups, except for the FCBHB2 sample, were clustered into five clades. This result indicated that the fungal composition in the samples in the same group was similar (Figure 4B). The PCoA and NMDS results demonstrated that the fungal communities in the same FCB group were similar, except for FCBHB2 and FCBAH2 (Figure 4C,D), indicating that the fungal communities in FCBXZ, FCBSC, and FCBQH groups were influenced by collection areas.

### 3.4. Interaction Analysis between Fungal Genera in FCB Samples

**Figure 5:**
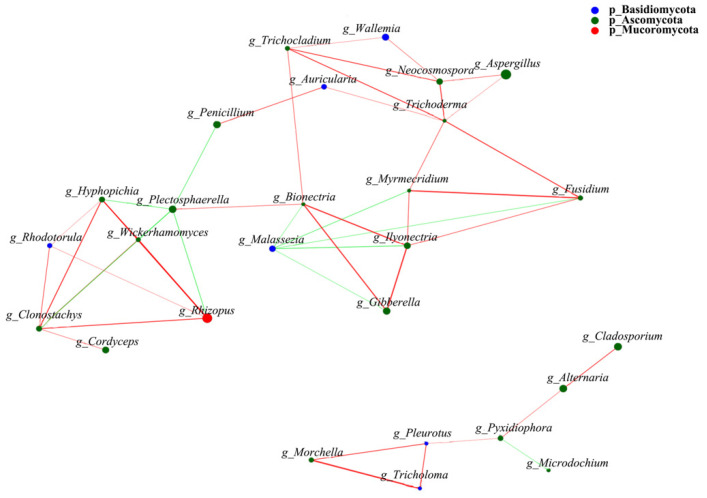
Network analysis with the Pearson correlations of the 30 top fungal taxa. The colors of the lines indicate positive and negative correlation, red indicates positive correlation, and green indicates negative correlation. The thickness of the line indicates the correlation coefficient. The thicker the line is, the higher the correlation between species is. The more lines, the more closely related the species is to other species.

The relationship between the 30 top abundant fungal genera was studied on the basis of the network result (Figure 5). A total of 11 negative correlations and 34 positive correlations were found among genera. *Aspergillus*, which was the most abundant genera in the FCB samples, was positively correlated with *Trichoderma* and *Neocosmospora*. *Rhizopus* was positively correlated with *Hyphopichia*, *Wickerhamomyces*, *Clonostachys*, and *Rhodotorula*, but was negatively correlated with *Plectosphaerella*. Meanwhile, *Cladosporium* and *Alternaria* showed a positive correlation with each other. *Malassezia* had the most negative correlations with other genera including *Bionectria*, *Gibberella*, *Ilyonectria*, *Fusidium*, and *Myrmecridium*. *Trichoderma* had the most positive correlations with six genera including *Auricularia*, *Myrmecridium*, *Fusidium*, *Trichocladium*, *Neocosmospora*, and *Aspergillus*.

## 4. Discussion

### 4.1. Fungal Community in the FCB Samples

FCB is a famous and precious TCM that has been applied in more than 200 Chinese patent medicines. Given its significant clinical effect, the quality and safety of FCB has attracted public attention. In recent years, studies about fungal contamination, which affects the safe utilization of TCM, have been widely reported in recent years. However, few studies comprehensively study fungal contamination in FCB. In the present study, we firstly applied DNA metabarcoding to study the fungal contamination in 15 FCB samples collected from the main producing areas in China. Meanwhile, we compared the differences in fungal community in five groups based on collection areas. In order to avoid the interference of endophyte, the elution method before DNA extraction was optimized. The surfaces of FCB samples were washed by PBS for six minutes. It has been reported that the endophytic fungal genera of medicinal plants mainly include *Chaetomium*, *Arbuscular*, *Botrytis*, *Leucocoprinus*, and *Lecanicillium* [21]. Our results showed that *Aspergillus*, *Rhizopus*, *Fusarium*, *Cladosporium*, and *Alternaria* were the dominant genera in the FCB samples excluding any endophytic fungi. The presence of these genera in other herbs has also been reported. In Brazil, Silva et al. investigated the occurrence of *Aspergillus* strains in yerba mate, and positively detected the *Aspergillus Nigri* section in all samples [21]. Zhu et al. firstly reported *Fusarium asiaticum* contamination in the stem of *Ligusticum chuanxiong*, which is a TCM that is commonly used for its therapeutic effect on cardiovascular and cerebrovascular diseases [22]. Chen et al. investigated fungal and mycotoxin contamination in 48 medicinal herb samples and found that 83.3% of the samples were contaminated with fungi. *Aspergillus* spp. and *Rhizopus* spp. were dominant at the species level, and three mycotoxins (aflatoxins, ochratoxin A, and citrinin) were detected in 39 samples [23]. Mycotoxin is a metabolite that is synthesized by mycotoxin-producing fungi under proper conditions. In the present study, five potential mycotoxin-producing fungi were identified including *Penicillium brevicompactum*, *P. citrinum*, *P. oxalicum*, *T. roseum*, and *A. restrictus*, which have been reported to be capable of producing mycotoxins in other studies. *Penicillium brevicompactum* is a common xerophilic fungus, which has the potential to produce mycophenolic acid (MPA) [24]. Overy and Frisvad also reported the *P. brevicompactum* contamination in ginger [25]. In their study, the *P. brevicompactum* contamination ratio reached 85%, and MPA contamination was positively detected in moldy ginger tissues. *P. citrinum* and *P. oxalicum* were two other *Penicillium* species that were detected in FCB samples. Citrinin and secalonic acid D were the main mycotoxins synthesized by these two fungi, respectively. These mycotoxins were not only harmful given their toxicity to the heart and liver system, but also caused cleft palates in humans [26,27]. *Trichothecium roseum* is a harmful postharvest fungus that may cause the spoilage of some fruits [28,29,30]. Furthermore, the T-2 toxin produced by *T. roseum* exhibits the highest toxicity in the trichothecene family, and can cause damage to cartilaginous tissues, apoptosis, and death [31]. *Aspergillus restrictus* is also a common potential toxigenic fungus of stored grains. It is capable of producing restrictocin, which inhibits protein synthesis [32]. Notably, the existence of potential mycotoxin-producing fungi is an essential prerequisite for mycotoxin contamination. Therefore, it is important to perform early detection of potential mycotoxin-producing fungi in herbs to prevent further mycotoxin contamination.

### 4.2. Relationship between Fungi and Herb in the Whole Production Chain

With the development of modern technology, increasing studies have demonstrated that fungi are closely related to the growth and development of medicinal plants during cultivation and harvest processes. Fungi also influence the quality and safety of herbal materials during processing and storage. Therefore, fungi play an important role in herbs in the whole production chain. It is essential to discuss the relationship between fungi and herbs in the production chain. The topics of soil rhizosphere and endophytic fungi in herbs have attracted global attention. Many researchers have demonstrated that these fungi not only affected the growth and development of medicinal plants, but also contributed to the synthesis of secondary metabolites. Wei et al. reported that the combined utilization of microbial inoculants and garbage enzymes improved the quality of *Salvia miltiorrhiza*. The accumulation of total tanshinones in *S. miltiorrhiza* was detected to increase by 40.45% after microbial inoculant treatment [33]. In Hungary, Engel et al. studied the effect of arbuscular mycorrhizal fungi (AMF) on the biomass, polyphenol profile, and content of three herbs. Their results showed that AMF inoculation remarkably promoted the yield of rosmarinic acid and lithospermic acid isomers in marjoram and lemon balm. They concluded that the application of AMF improved the quantity of these three herbs [34]. Shao et al. showed that three mycorrhizal fungi promoted the seed germination and growth of *Dendrobium chrysotoxum*, which is recognized as an endangered herb in China [35]. In addition, some fungi are necessary for the processing of herbs. Wang et al. monitored the changes in fungal community structure during the fermentation of *Shinkiku*, a traditional medicine for the treatment of anorexia and dyspepsia. Their results showed that *Aspergillus* sp. and *Rhizopus* sp. were the dominant fungal species in 13 *Shinkiku* samples from China and Korea. *Mucor* sp., *Saccharomyces* sp., *Xeromyces* sp., *Wallemia* sp., and *Fusarium* sp. were also detected in these samples. This study provided references for the stabilization of the quality of *shinkiku* through the analysis of the microbial community structure [36]. He et al. investigated the effect of processing methods on the quality of *Polygala tenuifolia*, indicating that processing decreased the fungal diversity and abundances, for example, some beneficial endophytic fungi. The lack of these beneficial fungi might cause fungal contamination in *P. tenuifolia*, and even mycotoxin contamination. Thus, He et al. concluded that fungi played a key role for ensuring the safety of *P. tenuifolia* [37]. Storage is an important stage wherein fungi probably affect the safety and quality of herbs. Citri Reticulatae Pericarpium (CRP) is a high-value TCM that normalizes spleen and stomach function effectively. The New Association of Geographical Indications recommends a typical storage time of three or more years for CRP, and states that long storage time improves the quality of CRP. Yang et al. indicated that CRP was easily contaminated by *Aspergillus* sp. and *Penicillium* sp. during storage. Interestingly, contamination resulted in an increase in the content of flavonoids, which were important medicinal components of CRP. Therefore, they speculated that the dominant *Aspergillus* and *Penicillium* fungi might be correlated with the quality of CRP [19]. In the present study, 34 fungal species were identified accurately in FCB. The function of some fungi has been described in previous studies. For example, *Sporisorium reilianum* and *Kabatiella zeae* have been reported as common pathogen fungi that inhibited the growth of plants [38,39]. In addition, *Pichia terricola* and *Hanseniaspora uvarum* were considered as novel candidate antagonists for the control of fungal contamination [40,41]. In South Africa, Amobonye et al. identified *Beauveria bassiana* as a new source of several industrial biocatalysts and biochemicals [42]. However, there were few studies on the function of these fungi in the production chain of FCB. Therefore, it was significant to analyze the relationship between fungi and FCB in its production chain in further research. Our work provides references and scientific basis for comprehensive studies about the effect of fungi on the quality of FCB.

### 4.3. DNA Metabarcoding Provides an Early Warning for Fungal and Mycotoxin Contamination

The fungal and mycotoxin contamination has become one of the herbal medicines’ main problems attracting global attention. Characterizing the fungal community, especially potential toxigenic fungi, may provide an early warning for subsequent potential mycotoxin biosynthesis. Recently, DNA metabarcoding has been applied to study fungal contamination in herbs. Jiang and collaborators [43] compared the difference in the fungal community between two TCMs—Morindae Officinalis Radix and Alpiniae Oxyphyllae Fructus through DNA metabarcoding technology. They indicated that *Penicillium* was the dominant genus in both herbal medicines. Thus, it was essential to pay more attention to preventing and control of the contamination of *Penicillium* fungi. Lu et al. used DNA metabarcoding to monitor the changes in fungal contamination and mycotoxin level during the malting process of barley. Results showed that the relative abundance of *Alternaria* increased during the malting process, and five potential mycotoxin-producing fungi were identified. Meanwhile, three *Alternaria* toxin residues were identified (alternariol, alternariol monomethyl ether, and tentoxin), concluding that inadequate malting conditions increase the fungal contamination frequency and mycotoxin production, especially the *Alternaria* fungi and their mycotoxins. Therefore, some strategies can be proposed, for example, fungicide treatment, in practical production [44]. Yu et al. assessed the fungal contamination in hawthorn and its processed products and observed that the relative abundance of *Alternaria* in dried samples was higher than that in roasted and charred samples. It was concluded that *Alternaria* fungi were the primary source of fungal contamination in dried hawthorn samples [45]. Based on these studies, DNA metabarcoding technology has been demonstrated as an efficient tool for evaluating fungal contamination in herbal medicines. This study used this technology to investigate the fungal community in FCB, a famous TCM in China. *Aspergillus*, *Rhizopus*, *Fusarium*, *Cladosporium*, and *Alternaria* were the dominant genera in FCB. All these genera were common contaminating fungal genera in herbs, which have been reported in previous studies. We further compared the differences in fungal community between FCB samples collected from different areas using hierarchical clustering analysis, principal co-ordinates analysis, non-metric multidimensional scaling analysis, Kruskal–Wallis H test, and LEfSe analysis, which were widely applied in the comparison of microbial community differences. We observed that the distribution of contaminating fungi in five groups was different. Notably, the average relative abundances of *Penicillium* and *Aspergillus* fungi were higher in the FCBHB group than in other groups. According to the collection information, the high temperature (30 °C) in Hebei province might provide a proper condition for the growth of some *Penicillium* and *Aspergillus* fungi. This result supports the conclusion proposed by Valencia-Quintana et al., that increasing environment temperature drove the fungal colonization in food [46]. Additionally, the FCBQH group presented the highest average relative abundances of *Cladosporium*. Similarly, Bullerman and collaborators also indicated that *Cladosporium* fungi grew well under adequate temperature conditions (<30 °C) [47]. In general, temperature is a key factor that affects the fungal community in FCB samples, and the differences in collection area temperature resulted in different dominant fungal genera in FCB samples. Thus, it is essential to control the storage temperature (<20 °C) to decrease the probability of fungal contamination.

## 5. Conclusions

In this work, we first revealed the fungal community in FCB, a famous herbal medicine in China, through DNA metabarcoding. While *Aspergillus*, *Rhizopus*, *Fusarium*, *Cladosporium*, and *Alternaria* were the dominant genera present in FCB samples, and five potentially toxigenic fungal species were also detected (*Penicillium brevicompactum*, *P. citrinum*, *P. oxalicum*, *Trichothecium roseum*, and *Aspergillus restrictus*). Meanwhile, the fungal community differences between groups based on collection areas were also observed. Our findings provide references for the target prevention of fungal contamination in FCB. Furthermore, it is essential to discuss further the role of the environment on the growth of fungi in the FCB practical production chain.

## Figures and Tables

**Table 1:** Collection information and accession numbers of FCB samples.

Sample No.	Sampling Location	Group	Collection Time	Collection Temperature	Genbank Accession No.
FCBXZ1	Tibet, China	FCBXZ	2021.08	23 °C	SAMN24255245
FCBXZ2	Tibet, China	FCBXZ	2021.08	23 °C	SAMN24255246
FCBXZ3	Tibet, China	FCBXZ	2021.08	23 °C	SAMN24255247
FCBQH1	Qinghai, China	FCBQH	2021.07	25 °C	SAMN24255248
FCBQH2	Qinghai, China	FCBQH	2021.07	25 °C	SAMN24255249
FCBQH3	Qinghai, China	FCBQH	2021.07	25 °C	SAMN24255250
FCBAH1	Anhui, China	FCBAH	2021.08	29 °C	SAMN24255251
FCBAH2	Anhui, China	FCBAH	2021.08	29 °C	SAMN24255252
FCBAH3	Anhui, China	FCBAH	2021.08	29 °C	SAMN24255253
FCBSC1	Sichuan, China	FCBSC	2021.08	30 °C	SAMN24255254
FCBSC2	Sichuan, China	FCBSC	2021.08	30 °C	SAMN24255255
FCBSC3	Sichuan, China	FCBSC	2021.08	30 °C	SAMN24255256
FCBHB1	Hebei, China	FCBHB	2021.08	30 °C	SAMN24255257
FCBHB2	Hebei, China	FCBHB	2021.08	30 °C	SAMN24255258
FCBHB3	Hebei, China	FCBHB	2021.08	30 °C	SAMN24255259

**Table 2:** Alpha diversity indexes of FCB samples.

Samples	Shannon	Chao	Coverage
FCBSC1	3.08	159.0	0.99998
FCBSC2	2.60	75.0	0.99997
FCBSC3	2.88	100.0	1.00000
FCBQH1	3.11	70.0	0.99996
FCBQH2	2.78	71.0	1.00000
FCBQH3	1.99	49.0	1.00000
FCBXZ1	1.79	22.0	1.00000
FCBXZ2	2.47	63.0	0.99998
FCBXZ3	2.76	63.0	0.99998
FCBHB1	3.16	186.5	0.99953
FCBHB2	3.25	96.0	0.99998
FCBHB3	3.30	199.5	0.99960
FCBAH1	3.52	109.0	0.99993
FCBAH2	2.21	12.0	1.00000
FCBAH3	1.66	143.7	0.99965

## Data Availability

The datasets presented in this study can be found in GenBank https://www-ncbi-nlm-nih-gov-s.webvpn.cams.cn/genbank/ (accessed on 20 December 2021).

## Supplementary Materials

The following supporting information can be downloaded at: https://www.mdpi.com/article/10.3390/jof8080876/s1, Figure S1: Rarefaction curve analysis of 15 FCB samples; Figure S2: Venn analysis of shared and unique OTUs in different FCB groups; Table S1: OTU distribution in 15 FCB samples; Table S2: Detailed information about fungal taxa at the species level.
